# Surgical correction of persistent truncus arteriosus on a 33-year-old male with unilateral pulmonary hypertension from migration of pulmonary artery band

**DOI:** 10.1186/s13019-016-0435-x

**Published:** 2016-03-29

**Authors:** Wen Ruan, Yee Jim Loh, Kenneth Wei Qiang Guo, Ju Le Tan

**Affiliations:** Department of Cardiologoy, National Heart Centre Singapore, Level 12, 5 Hospital Drive, Singapore, 169609 Singapore; Department of Cardiothoracic Surgery, National Heart Centre Singapore, Level 12, 5 Hospital Drive, Singapore, 169609 Singapore

**Keywords:** Persistent truncus arteriousus, Truncal valve regurgitation, Unilateral pulmonary hypertension, Case report

## Abstract

**Background:**

Persistent truncus arteriosus is a rare congenital condition with which survival into adulthood is dismal without surgery. This is the oldest patient reported to our knowledge demonstrating the feasibility of assessing operability in persistent truncus arteriosus with unilateral pulmonary stenosis, and performing full corrective surgery in adulthood.

**Case presentation:**

We report a Chinese male with successful correction of Type I persistent truncus arteriosus at 33 years of age. He had unilateral pulmonary hypertension from migration of pulmonary artery band from the main to the right pulmonary artery, severe truncal valve regurgitation from previous infective endocarditis, and progressive congestive heart failure. Improvement of lung perfusion was demonstrated 21 months post operation.

**Conclusion:**

This case demonstrated that in patients with persistent truncus arteriosus and two pulmonary arteries, pulmonary vascular disease or underdevelopment of one lung does not preclude a full corrective surgery so long as the other vascular bed is normal. It is important to emphasize the importance of assessing patient’s operability in totality.

## Background

Persistent truncus arteriosus (PTA) is a rare congenital condition with normal atrial arrangement and concordant atrioventricular connections in which a solitary arterial trunk arises from the base of the heart and supplies the coronary, pulmonary and systemic arteries [1]. It accounts for 1.2–3 % of all congenital heart malformations. Before the surgical era, children with TA demonstrated 80 % mortality in the first year of life [2]. If repaired in early infancy, the long term results reported in 1997 were 90 % at 5 years, 95 % at 10 years and 85 % at 15 years [3]. If left uncorrected, patients usually do not survive or develop irreversible pulmonary vascular disease. Rare survival of uncorrected type I PTA into childhood or adulthood were only seen in isolated case reports [4, 5].

Pulmonary artery banding, on main or both PA, was a temporary measure to control congestive heart failure, often with a view to perform full corrective surgery at an older age. In the earlier days, surgical mortality was high at 55 % for neonates, 44 % for infants 1–3 months old, and 2 % for patients older than 6 months [6]. However, the PA band may migrate over time, especially in type I TA, leading to obstruction of one PA and development of pulmonary vascular disease in the other; or there may be failure of PA growth distal to the band or distortion of the PA. Hence, this is now reserved as a palliation for patients not suitable for definitive repair. We presented the result of a corrective surgery on an adult with type I PTA, unilateral pulmonary hypertension due to migration of PA band, and severe truncal valve regurgitation. He is thus far the oldest type I PTA in literature whom made good recovery with improvement in lung perfusion post operation.

## Case presentation

**Fig. 1:**
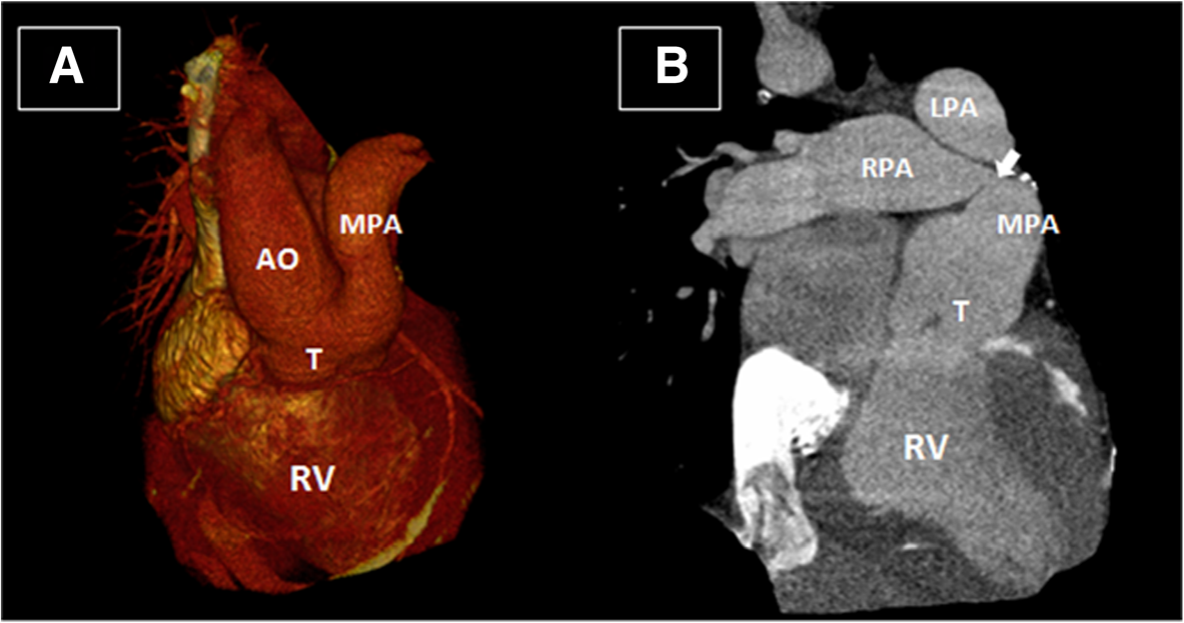
Computed tomography of the chest. The heart size is enlarged. On the 3-dimensional volume rendered image (**a**), there is a common trunk of the ascending aorta and main pulmonary artery (PA). The aortic arch is right-sided and the pulmonary arteries are grossly dilated. On the coronal view (**b**), the tight narrowing at the beginning of right PA (white arrow) is where the migrated PA band from main PA locates. T: truncus; MPA: main pulmonary artery; LPA: left pulmonary artery; RPA: right pulmonary artery; AO: aorta

A 33-year-old male was diagnosed to have type I truncus arteriosus (TA) during infancy. He underwent palliative main PA banding at the age of four months. No following corrective surgery was performed at his family’s choice. He had an uneventful childhood. At 26, he presented with *Streptococcal Gordonii* endocarditis with severe truncal valve regurgitation. This was complicated with septic emboli to his lungs and kidneys. At the same time, he was found to have left sided pulmonary hypertension due to migration of the PA band to the right PA (RPA) on the computed tomography of the chest (Fig. 1). He survived from a stormy hospital stay which required intubation and mechanical ventilation, and completed 5 weeks of antibiotics. A decision was made not for surgery at that point of time in view of the high surgical risk and uncertain surgical outcomes at this age [7].

He remained relatively well in New York Heart Association (NYHA) functional class I for 3 years, subsequently, his functional class deteriorated to NYHA II. On annual echocardiographic follow up, there was evidence of increase in size and decrease in systolic function of the left ventricle (LV). At 32 years old, he was hospitalized for worsening heart failure. On physical examination, he was thinly built with pectus carinatum, finger clubbing, raised jugular venous pressure and pitting edema over bilateral lower limbs. He had displaced apex beat, first and second heart sounds were present. There were ejection systolic murmur and early diastolic murmur at the left parasternal edge. The oxygen saturation was 90 % on room air. Blood investigation were unremarkable except for elevated hemoglobin of 17.9 g/L and serum creatinine of 105 mmol/L. Electrocardiogram revealed right ventricular (RV) hypertrophy with strain pattern. Chest radiograph showed an enlarged heart with prominent pulmonary trunk.

**Fig. 2:**
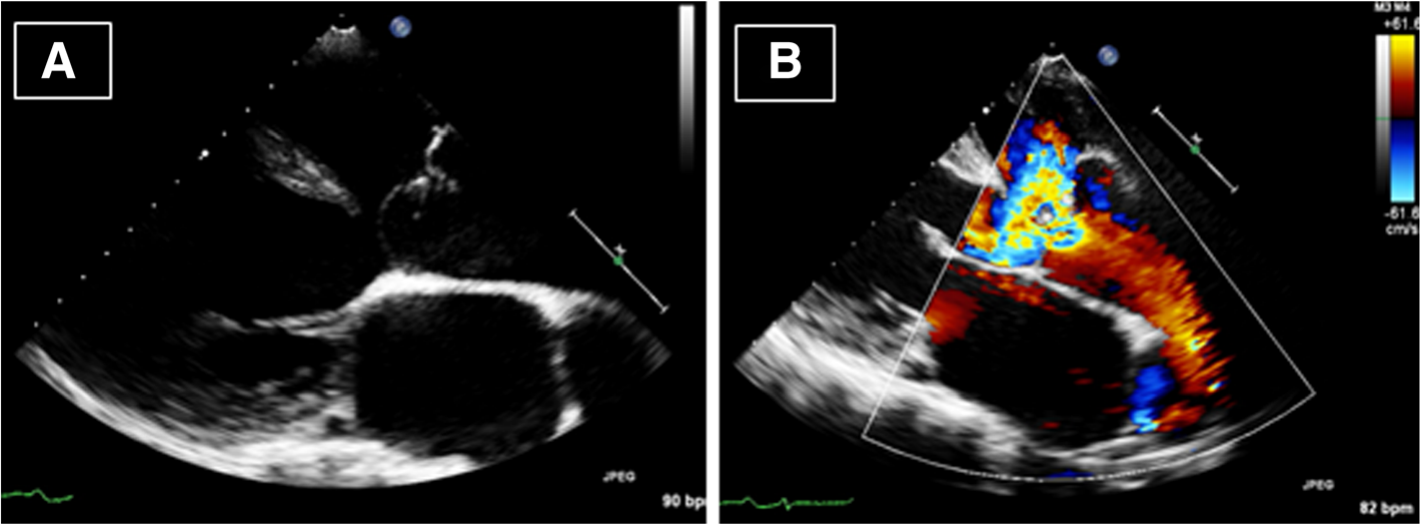
Echocardiogram showing overriding truncus arteriosus (TA), truncus valve (TV) and a large unrestrictive outlet ventricular septal defect (VSD) (**a**). Color Doppler showing severe truncal valve regurgitation (**b**)

**Fig. 3:**
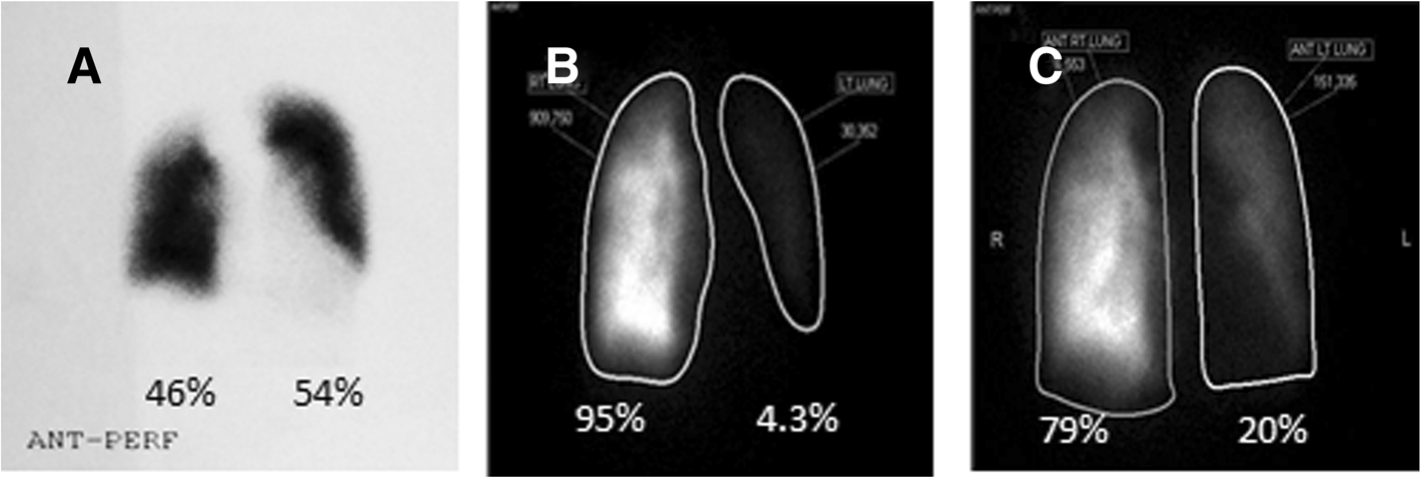
Technetium-labeled (Tc-99 m) macroaggregated albumin (MAA) lung perfusion scan, evaluates how well blood circulates within the lungs. It showed marked decrease in perfusion to the left compared to the right lung (4.3 % vs. 95.7 %) prior to operation (**b**), while both lung perfusions were almost similar (54 % vs 46 %) 6 years prior (**a**). This is because the right lung was protected by the RPA band, while the left lung received the majority of cardiac output, exposing it to significant increased flow and pressure. As a result, there was increased pulmonary vascular resistance and poorer delivery of the radioactive tracer to the left lung [17, 18]. In this case, pulmonary vascular remodelling was shown to be partially reversible even operated late. Twenty-one months post operation, a repeat lung perfusion scan showed improved left lung perfusion to 20.2 % (**c**)

Trans-thoracic echocardiogram showed type I TA (Fig. 2). The LV was severely dilated with ejection fraction of 40–45 %. The truncal valve annulus was dilated with severe regurgitation. The RV systolic pressure was 116 mmHg with severe RV hypertrophy. Invasive haemodynamic study revealed progression of pulmonary vascular disease compared to 6 years ago. (Table 1) However, the RPA was still protected with no significant rise in PA pressure. Calculated left and right pulmonary vascular resistances (PVR) were 10.2 Wu and 2.6 Wu respectively. At the same time, lung perfusion scan showed marked deterioration of perfusion activity to the left lung (4.3 % vs 54 %) compared to 6 years ago (Fig. 3a and b).

**Table 1:** Haemodynamic Data Obtained during Cardiac Catheterization and Cardiac MRI

	Year 2006		Year 2012 (pre-op)	
	Pressure (mmHg)	Oxygen Saturation(%)	Pressure (mmHg)	Oxygen Saturation(%)
Superior vena cava	Mean 6	68.4	Mean 5	62.1
Inferior vena cava	Mean 6	64.9	Mean 4	70.5
Mid right atrium	7/7/6	62.6	7/5/4	60.2
Right ventricle	83/4/7	61.6	102/3/11	55.6
Left ventricle	110/3/14	83.3	104/8	74.3
Truncus	102/41/70	81.7	110/40/68	89.4
Main pulmonary artery	108/48/72	83.5	108/40/53	88.9
Left pulmonary artery (LPA)	89/46/66	81.2	110/34/67	88.1
LPA flow (fr MRI), ml/s				98
LPA PVR (fr MRI)^a^, Wu				6.62
Right pulmonary artery (RPA)	20/14/16	86.7	24/16/20	89.8
RPA flow (fr MRI), ml/s				67
RPA RVR (fr MRI), Wu				1.97

Pressure data are expressed as systolic, diastolic and mean pressures, respectively. PVR, pulmonary vascular resistance

^a^LPA PVR = mean LPA pressure-LVEDP/flow LPA

In view of the worsening symptoms, cardiac size and function, as well as progression in asymmetrical pulmonary vasculature remodeling, decision was made for total correction at the age of 33. Surgery was performed via a standard median sternotomy and pericardiotomy. The establishment of cardiopulmonary bypass included ascending aorta, superior and inferior vena cava cannulation and core cooling to 25 °C. Circulatory arrest was achieved by fibrillating the heart. The bilateral branch PAs were snared at the commencement of cardiopulmonary bypass and aortic cross-clamping was applied. Truncus was transected, and the PA orifice and the coronary arteries were identified. A single dose of direct antegrade cardioplega (custodial) was introduced down the coronary ostium with spontaneous cardiac arrest.

Intraoperative findings were consistent with imagings: The truncal valve was tricuspid, the leaflet was damaged beyond repair by the endocarditis. The truncus root was very dilated and overriding into the RV (>50 %). The left coronary artery (LCA) orifice originated at a higher level than the right coronary artery (RCA) and had a short intramural course, arising from the posterior facing sinus. RCA arised from the anterior facing sinus. No abnormal course of the LCA.

The coronary buttons were cut out. Left ventricular outflow tract was established by a valve conduit (size 31 mm, Metronic, Inc, Minneapolis, MN) and the coronary buttons were anastomosed. The ventricular septal defect (VSD) was closed with Dacron patch. The RV to PA continuity was established by aortic homograft conduit (size 25 mm). As the stenosis of RPA from previous PA band was at the orifice of the RPA, this was dealt with during the suturing of the RV-PA conduit. Autologous pericardial “hood” was used to augment RV outflow tract to homograft anastomosis proximally. The distal end of the homograft was cut at an angle and then incised to enlarge the anastomotic suture line. Prior to completion, a trans-esophageal echo was performed to detail the VSD closure, bileaflet prosthesis function, and patency of PV-PA conduit with minimal regurgitation.

Challenges associated with operation: 1) Bleeding. Patient developed massive bleeding originating from the Bental anastomosis, required going back on-pump and reinforcing the suture line towards the end of the surgery. Chest was kept open for 48 h with the support of intra-aortic balloon pump (IABP) until day 4 post operation. 2) Arrhythmias. After removal of IABP, the patient developed atrial fibrillation and was started on intravenous amiodarone. He then developed ventricular standstill and emergency arterio-venous Extracorporeal Membrane Oxygenation (ECMO) had to be inserted. He made good recovery after ECMO was explanted on day 7 post operation.

Patient was followed up after discharge and remained in NYHA II. At 21 months after the primary procedure, lung perfusion scan suggested improvement of the left lung perfusion activity from 4.3 to 20.2 % (Fig. 3c).

## Discussion

There were several challenges when approaching this case: Firstly, truncal valve regurgitation is the major life-threatening complication of truncus arteriosus. It affects postoperative outcomes in patients with this cardiac anomaly. In our case, due to severe truncal valve damage and annular dilatation (>40 mm), it was difficult to find a suitable prosthesis (largest size 37 mm). Although several truncal valve reparative techniques have been reported, it remains challenging due to the unsatisfactory results [8]. 2) Origin of the truncal valve predominantly from the RV, causing under development of the aortic arch and preferential flow into PAs over that into the aorta. This is associated with a higher risk of creation of subaortic stenosis post-surgery. 3) Plicating the aortic root to suit the prosthesis size will almost require a root remodelling surgery with plication done right down to the base of the root. In such cases, the valves should be normal for maximal benefit. However the truncal valve was damaged from previous infective endocarditis. Also as the patient is very young. Plication of the root may run a risk of future aortopathy and dilation requiring a re-operation. Hence, we decided that an isolated truncal valve repair was not a long term solution for this patient. Taking into consideration, there is still unresolved unilateral pulmonary hypertension and progression of pulmonary vascular disease.

Secondly, there was no combined heart-lung transplant program available in our country. A ventricular assist device was not helpful due to uncorrected VSD, RV failure and raised pulmonary vascular resistance. However, the long term results of full PTA corrective surgery at a later age (above 1 year old) are largely unknown.

A thorough search of literature revealed a few small series of surgical repair in older children, and isolated case reports in adults. Marcelletti et al. reported a series of 96 patients with surgical repair of TA (73 % type I TA, 18 % has unilateral PA stenosis by nature or banding) from 1967 to 1975. The 30-day mortality for those underwent repair after 2 years old were about 21 %. The relation between pulmonary resistance and surgical risk was only studied for those with presence of both right and left PAs, and it was higher in patients with indexed PVR (iPVR) >8 Wu.m2 pre-operatively [9]. Stegmann et al. reported 8 cases of type I TA repair in patients’ age from 2 month to 4.5 years (2 of which had PA banding before) from 1976 to 1981. Only one 3.5-year-old child died postoperatively because of RV failure. In this child the iPVR had risen to 13 Wu.m2 despite PA banding [10]. In recent years, Talwar et al. reported 9 children with PTA (7 with type I PTA). Among the 9 patients, 2 had PA stenosis (1 at the origin of PA trunk from the TA and the other at the origin of RPA). These 9 patients underwent corrective surgery at mean age of 3 (range, 1 to 12 years) between 2000 and 2012. The hospital mortality was 22.2 % (2 deaths of Type 1 TA, 1 due to intractable pulmonary arterial hypertension and 1 due to sepsis). The rest had good functional recovery and no mortality after a mean 39 months (range, 3 month to 138 months) follow up [11]. Zhang et al. reported 12 patients (83 % Type 1 PTA) with only 1 patient having PA stenosis. They underwent repair of PTA at mean age 6.4 (range 1.2 to 19 years) between 2006 and 2010. There was no early mortality or requirement of reoperation after a mid-term follow up of mean 2.4 years (1.1 to 5.3 years). All patients had iPVR of less than 8 Wu.m^2^ [12]. These findings suggested that late correction of TA should be undertaken prior to the development of pulmonary vascular disease. On the other hand, successful surgical correction in adults were only reported in a 31-year-old male with PTA and severe pulmonary vascular obstructive disease [13], and a 28-year-old male with atypical PTA (The artery to the middle lobe of right lung originating from the descending aorta with stenosis at the origin). Although there were severe hypertension in the right chambers (100/50/70 mmHg), his lung was protected until adulthood due to the pulmonary trunk stenosis at the origin [14].

Thirdly, difficulty in assessment of operability: With truncus arteriousus defects, the possible inequalities of pressure and flow between the two PAs made precise calculation of pulmonary resistance difficult. To solve this problem, a combined right heart catheterization for pulmonary pressures, and a cardiac MRI for flow in main and branch PA was used in this case. As pressure distal to the PA band was not elevated, pulmonary vasoreactivity test was not performed. In the era when cardiac MRI was not available, McFAUL et al. presented 27 patients with TA and previous PA banding (range,6 weeks to 14 years). They managed to demonstrate that for patients who had two PAs, the presence of at least one adequate PA having low distal pressure or arteriolar resistance is the minimal criterion on which operability is based [15].

## Conclusions

This case demonstrated that in adult PTA patients with two PAs, pulmonary vascular disease or underdevelopment of one lung does not preclude a full corrective surgery so long as the other vascular bed is normal. It is important to emphasize the importance of assessing patient’s operability in totality. This patient is the oldest PTA patient reported in literature to have undergone a complete repair at the age of 33, having had relatively protected right lung vasculature in infancy by migration of a proximately placed PA band. This, together with the patient’s improvement of lung perfusion scan postoperatively, is in congruent with other reports of successful corrective surgery in older PTA patients with either bilateral [9] or unilateral PA [16].

## Consent

Written informed consent was obtained from the patient for publication of this case report and an accompanying images. A copy of the written consent is available for review by the Editor-in-chief of this journal.

## Abbreviations

ECMO: extracorporeal Membrane Oxygenation
IABP: intra-aortic balloon pump
LCA: left coronary artery
LV: left ventricle
PA: pulmonary artery
PTA: Persistent truncus arteriosus
PVR: pulmonary vascular resistance
RCA: right coronary artery
RV: right ventricle
TA: Truncus arteriosus
VSD: Ventricular septal defect
